# Remote Monitoring in the Home Validates Clinical Gait Measures for Multiple Sclerosis

**DOI:** 10.3389/fneur.2018.00561

**Published:** 2018-07-13

**Authors:** Akara Supratak, Gourab Datta, Arie R. Gafson, Richard Nicholas, Yike Guo, Paul M. Matthews

**Affiliations:** ^1^Data Science Institute, Imperial College London London, United Kingdom; ^2^Division of Brain Sciences, Department of Medicine, Imperial College London, London, United Kingdom; ^3^Charing Cross Hospital, Imperial College Healthcare Trust, London, United Kingdom; ^4^UK Dementia Research Institute, Imperial College London, London, United Kingdom

**Keywords:** multiple sclerosis, real world data, biomarkers, gait, actigraphy, remote sensing technology

## Abstract

**Background:** The timed 25-foot walk (T25FW) is widely used as a clinic performance measure, but has yet to be directly validated against gait speed in the home environment.

**Objectives:** To develop an accurate method for remote assessment of walking speed and to test how predictive the clinic T25FW is for real-life walking.

**Methods:** An AX3-Axivity tri-axial accelerometer was positioned on 32 MS patients (Expanded Disability Status Scale [EDSS] 0–6) in the clinic, who subsequently wore it at home for up to 7 days. Gait speed was calculated from these data using both a model developed with healthy volunteers and *individually personalized* models generated from a machine learning algorithm.

**Results:** The healthy volunteer model predicted gait speed poorly for more disabled people with MS. However, the accuracy of individually personalized models was high regardless of disability (*R*-value = 0.98, *p*-value = 1.85 × 10^−22^). With the latter, we confirmed that the clinic T25FW is strongly predictive of the maximum sustained gait speed in the home environment (*R*-value = 0.89, *p*-value = 4.34 × 10^−8^).

**Conclusion:** Remote gait monitoring with individually personalized models is accurate for patients with MS. Using these models, we have directly validated the clinical meaningfulness (i.e., predictiveness) of the clinic T25FW for the first time.

## Introduction

Tools for the sensitive assessment of disability and its progression in patients with multiple sclerosis (MS) are needed (1). A major challenge has been to define measures that meaningfully reflect the concept of interest and are sensitive to change over time for the full range of disability. The most commonly accepted measure to quantify the disability in MS is the Expanded Disability Status Scale (EDSS) (2). However, the EDSS shows variable sensitivity to change with level of disability.

Walking speed has been used as an additional, objective outcome measure of performance related to disability (3–5). Walking difficulties are strongly correlated with progression of MS (6, 7) and reduced quality of life (5, 8, 9). At present, walking speed is evaluated only in the context of short test walks in the clinic standardized as the Timed 25-Foot Walk (T25FW) or the Six-Minute Walk (6MW) (10). Even though these tests are believed to be reliable measures, the extent to which the walking speed measured during performance tests in the clinic correlates with unconstrained walking behavior at home is unknown. Determining this is fundamental to appreciating the meaningfulness for the patient of gait speed measures in the clinic and they are subject to potential interpretative concerns. For example, clinic measures are typically sparsely acquired (a single, short time period in the day during an assessment visit) in an atypical environment. They also may be subject to time-of-day influences such as fatigue (5, 11–13).

Mobile actigraphy is promising as a reproducible, quantitative approach to remote assessment of walking speed for people with MS (5, 14, 15). Many recent studies have utilized single or multiple actigraphy devices worn on the wrist or waist to collect gait-related data (4, 16–19). Walking speeds can be estimated from actigraphic data using models that relate features derived from the timing of successive accelerations of the accelerometer(s) attached to the body and individual steps (16). Actigraphy can be used readily to assess movement in the home environment over extended periods.

However, there are three major limitations to existing approaches. Firstly, some of them have used the gait data from wrist-worn devices. Movement at the wrist is influenced by factors other than gait. This can lead to high walking speed estimation errors (20, 21). Greater accuracy can be achieved by attaching a device close to the center of mass of the body in order to reduce influences of limb movements unrelated to walking. In controlled environments, a waist-worn position outperforms the wrist-worn position in accuracy of counting the number of steps (20, 21). Secondly, most of the approaches described thus far have used actigraphic data from a limited number of MS or healthy-control subjects, along with a limited amount of associated demographic information (e.g., weight, height and step length) to build generic healthy volunteer models for walking speed estimation. However, because gait biomechanics change with body habitus and disability and can have a large influence on acceleration patterns across a step, the accuracy of generic models can vary substantially between individuals (7, 22). Approaches developed using models applicable to healthy volunteers fail particularly when estimating the walking speed in patients with greater levels of disability (16).

Here we describe an automated, machine learning approach for individualized actigraphy model calibration that allows accurate, personalized walking speed estimation for patients with MS. We use software based on this personalized model to measure walking speed remotely in the home environment. We demonstrate that gait speed can be measured accurately, remotely and for extended periods across a wide range of disability. With these data, we performed the first direct validation of speeds from the standard clinical T25FW relative to patients' maximum sustained walking speeds in their home environments.

## Methods

### Participants

We recruited participants from the MS clinics of Imperial College Healthcare Trust (ICHT) between 1 April 2015 and 31 March 2017 (Table 1) (RN). The participants provided demographic information and then underwent a screening evaluation for generating an EDSS score. Criteria for inclusion were clinically-supported diagnosis of MS, an EDSS score of 0.0–6.0 at the screening evaluation and an ability to understand and follow the study instructions. During periods in which actigraphy data was acquired, a device was attached in a paraspinal position on the lower-back using surgical tape. The clinical study protocol was approved by the London Bromley National Research Ethics Committee (14/LO/0292). All participants provided written informed consent before beginning the study.

**Table 1:** Patient demographic data and mean baseline times (*n* = 8 trials) for the Timed 25 Foot Walk (T25FW, in seconds) and gait speeds calculated from these (meters/second).

**Patient**	**EDSS**	**Age**	**T25FW (s)**	**Gait speed 25 FW (m/s)**
1	2	40–45	5.0	1.5
2	2	30–35	6.9	1.1
3	5.5	40–45	10.1	0.8
4	1	30–35	6.0	1.3
5	5	45–50	10.2	0.8
6	2.5	30–35	6.2	1.2
7	3	35–40	6.7	1.1
8	2.5	35–40	7.5	1.0
9	3	50–55	7.9	1.0
10	2	40–45	5.3	1.5
11	6	45–50	9.8	0.8
12	1	40–45	9.0	0.9
13	2	25–30	5.6	1.4
14	1.5	30–35	8.7	0.9
15	4	45–50	6.7	1.1
16	3.5	50–55	7.3	1.0
17	4	30–35	7.5	1.0
18	6	50–55	8.4	0.9
19	2	30–35	6.1	1.3
20	1	45–50	4.8	1.6
21	2.5	35–40	6.0	1.3
22	1.5	30–35	6.8	1.1
23	6	45–50	14.1	0.6
24	6	40–45	9.7	0.8
25	6	45–50	18.3	0.4
26	6	55–60	10.5	0.7
27	1	25–30	4.4	1.7
28	5.5	30–35	9.3	0.8
29	5.5	35–40	7.1	1.1
30	1.5	25–30	7.5	1.0
31	1	25–30	5.7	1.3
32	1	45–50	5.5	1.4

*Ages expressed to nearest 5 years to preserve anonymity*.

Briefly, the study design included three stages for the participants. For the first two stages, an actigraphy device was positioned on the lower back of each particiant with surgical tape before timed 25 foot walk (T25W) testing and supervised 6 min free walking in an open hospital clinic corridor (GD, AG). For the third stage (which volunteers could consent to or not), the volunteers then were asked to continue wearing the device for up to 7 days as they want about their usual daily activities in their home (“real world” environments) (AS, AG). Timed data was accumulated in each device for the entire period. The devices then were returned to the clinic directly by each volunteer or through the post. Data was downloaded from each device onto a server (AS). The analysis plan involved first testing of the relationship between measured gait speed and that predicted from analysis of actigraphy data using a model optimized for healthy volunteers (AS). A personalized gait model then was developed for each subject, trained using data acquired in the clinic, and tested using clinic gait data not used for the model development. Finally, the personalized model was applied to analysis of data acquired from the “real world” data and the maximum sustained “real world” gait speed determined in these data was compared with the T25W data acquired in the clinic. This was an exploratory study intended to estimate and compare measures and was not formally powered.

### Actigraphy devices

An AX3-Axivity tri-axial accelerometer protected with silicone backing was used in this study. An internal battery provides power for up to 14 days of use. The accelerometer measures acceleration forces from three dimensions in units of gravitational force (g-force, approximately 9.81 m/s^2^).

AX3-GUI software (https://github.com/digitalinteraction/openmovement/wiki/AX3-GUI) provided by Axivity was used to configure and download raw acceleration data from the accelerometer. This software was also used to convert raw acceleration data into comma-separated-value (CSV) files. Each row in the CSV files consists of the timestamp and the g-forces from vertical, horizontal and forward directions (referenced to the Earth's gravitational field). These CSV files were used to calibrate the subject-specific model for walking speed estimation, which will be discussed in the next section.

### Data collection

Each volunteer was asked to complete two phases of data collection in the clinic: one for model calibration and another for model validation in the clinic. Some subjects agreed to a third phase of data collection by wearing the Axivity device as they went about usual “real world” activities of daily life in their home environments. Acceleration data from the calibration phase were used to build subject-specific models for walking speed estimation. The data from the validation phase—in which gait speeds were both directly measured in the clinic and estimated using the personalized models—were used to evaluate the performance of the personalized model relative to the earlier approach. The data from the home monitoring were used to test how well the clinic timed 25-foot walking speed predicted maximum sustained walking speeds of the MS volunteers in the home environment.

During the data collection in the clinic, a researcher walked alongside the volunteer to record the time and distance walked (meters) using a measuring wheel. The speed of each walk was calculated as distance (meters)/time (seconds). At the conclusion of each participant's involvement with the study, they were asked to provide feedback about the comfort and tolerability of the device.

During the *calibration* phase, volunteers repeated a Timed 25-Foot Walk (T25FW) 8 times (Table 1), as well as a 2-Minute Timed Walk (2MIN) 2 times (Supplementary Table 1). A separate supervised walk was performed following these to provide data for *validation* of the model. Some subjects agreed to wear the device at home for up to 7 days (including during sleeping, if tolerated).

#### Subject-specific gait calibration

For each subject, the data collected during the calibration phase were used to build a subject-specific model for walking speed estimation. There were four main steps taken with the raw actigraphy data for development of this model: (i) preprocessing; (ii) extraction of data related to individual steps taken; (iii) identification and measures of specific features associated with individual steps; and (iv) calibration of the model.

The pre-processing step transformed the raw acceleration data acquired on the Axivity device into a format that could be used for feature extraction. The first stage involved segmenting the time-series of acceleration data acquired into individual periods of walking. This walking data was then tilt-adjusted (23) to account for the arbitrary tilt of the accelerometer relative to the Earth's gravitational field. Next, a low-pass filter (fourth-order, zero-lag Butterworth filter at 20 Hz) was applied to remove noise and higher frequency artifacts not related to gait.

In the second stage of the calibration, this pre-processed data from continuous periods of walking was further segmented into that describing individual steps based on detection of the peak of forward acceleration preceding a change of the sign from positive to negative acceleration marking contact of the foot with the ground at the start of the step (24) (Supplementary Figure 1). Twenty-nine characteristic features were then measured automatically for each step (16) (Supplementary Table 2).

With the full set of features generated from the calibration walking tests performed by each subject in the clinic, Support Vector Regression (SVR) (25) was used to build a personalized model to calculate gait speed from the actigraphy data (see Supplementary Methods).

A generic model using similar data acquired from healthy volunteers was developed in the same way except that step features aggregated from a group of healthy volunteers were used to develop the model rather than using only data from a single individual.

### Data analyses

The relative accuracy of models was compared from mean differences between the gait speeds measured directly by the investigator and those estimated by the models. To test for systematic bias in the estimation errors with walking speed or disability (EDSS), we used Bland-Altman plots (26) of estimation errors relative to either the directly measured walking speed or EDSS. We tested for relationships between EDSS and gait speed using Pearson's R coefficient (27).

To explore differences in step characteristics between healthy volunteers and patients with MS in an effort to better understand differences in performance of generic healthy volunteer and personalized models, we used Principal Components Analysis (PCA) (28). For this exploratory analysis, we extracted step features from walking data of both patients with multiple sclerosis and from previously acquired healthy volunteer data during T25FW and applied PCA to compute the first and second principle components, which were then compared graphically between the groups.

Twenty two of the participants with MS wore the Axivity devices at home. Accelerometry data was acquired continuously during periods that the device was worn. From these data, periods of sustained (>25 feet) walking during daytime hours were identified from the full actigraphy time series data for each subject and the gait speeds were calculated using personalized gait models. Both the *maximum* sustained walking speed over the full period of remote observation and the *mean* of gait speeds measured during periods of sustained walking were calculated. The relationship of the maximum sustained walking speed in the home environment to the mean gait walking speed measured during T25FW in the clinic was tested using Pearson's R coefficient (27).

### Patient reports of device comfort

Each subject was asked at the end of their participation in the study if they found wearing the device comfortable (yes or no responses). Narrative feedback concerning any sources of any discomfort or other concerns was solicited.

## Results

### Patient personalized models for walking speed improve accuracy relative to a generic model

There were 32 MS volunteers (15 men and 17 women; mean age, 39.9 ± 8.6 years; median EDSS score, 2.5, range 1.0-6.0) recruited into the study. All 32 MS volunteers used an actigraphy device in the clinic and 22 volunteers (11 men and11 women, age: 39.8 ± 8.9 years) agreed to wear a device for up to 7 days in their home environments. The six volunteers with the greatest disability (EDSS, 6.0) used single-point walking aid devices (stick or crutch). Data from all of the patients were compared to previously collected data from seven healthy volunteers whose data was used to calibrate a healthy population gait model (see Supplementary Methods).

Most prior approaches (and those most commonly used with commercial devices) have applied generic models for estimation of gait speeds from actigraphy. We tested whether a patient personalized gait model could more accurately estimate gait speed than a generic model based on gait characteristics of healthy volunteers. To do this, we estimated walking speed for the MS patients based on actigraphy measures using either a model derived from gait characteristics of the healthy subjects (generic model) or one developed individually for *each* of the patients based on the direct observations in the clinic (personalized model).

**Figure 1:**
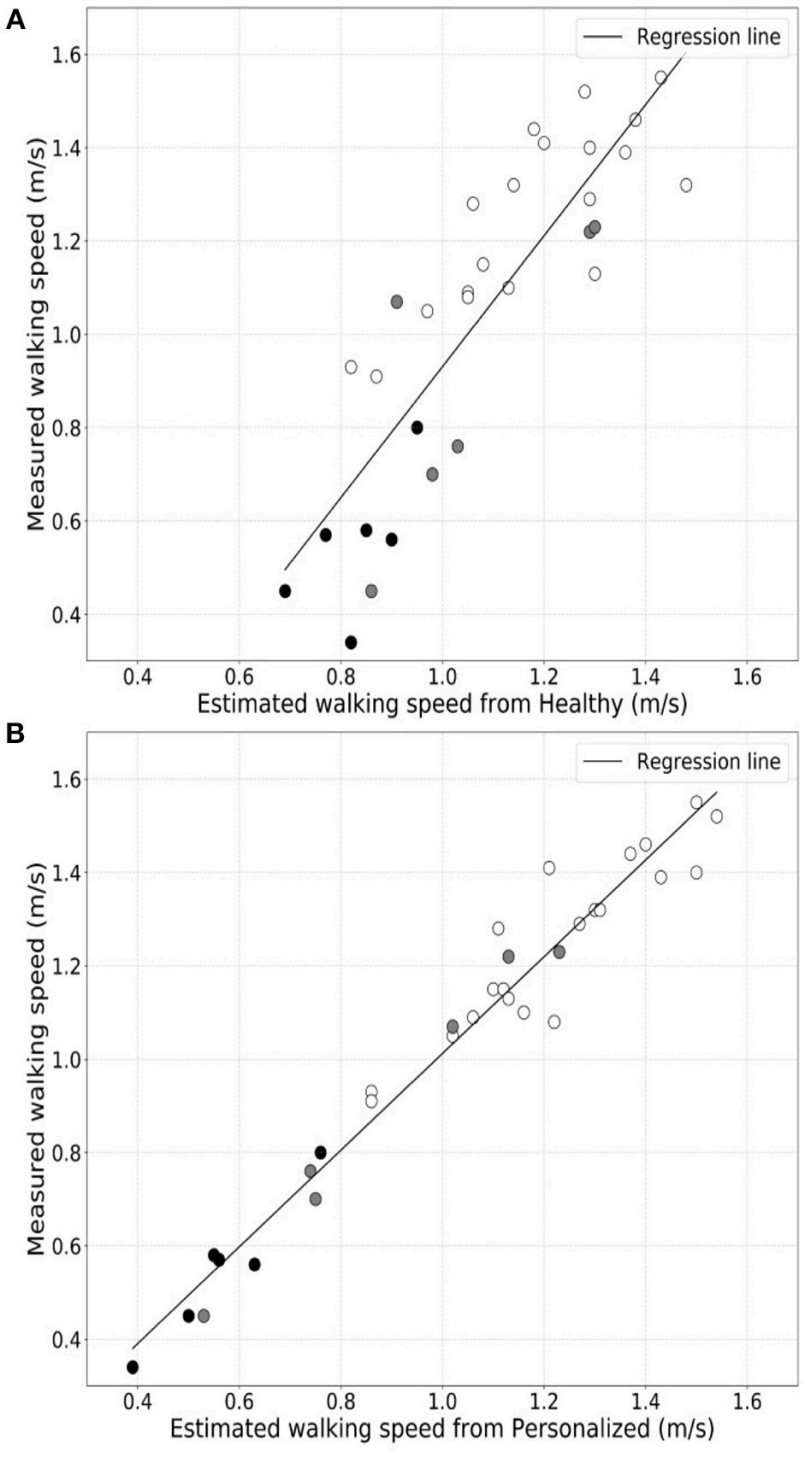
Comparison of correlations between directly measured walking speeds for the full group of patients and either that estimated from actigraphy data using a generic population **(A)** or personalized models **(B)**.

**Figure 2:**
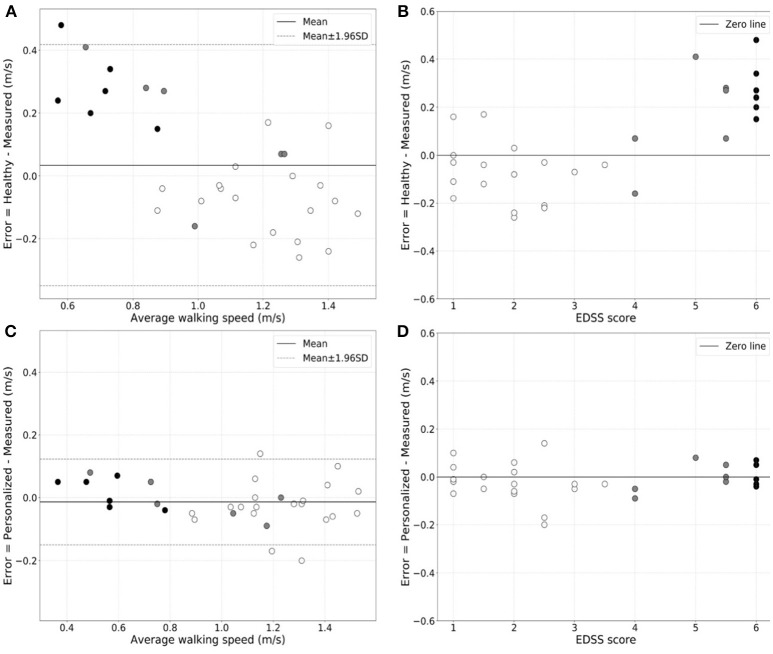
Bland-Altman plots of the variation in error in walking speed estimation from actigraphy relative to the directly measured walking speed as a function of the walking speed for the generic population **(A)** or personalized **(C)** models. The white-filled, gray-filled circles, and black-filled circles denote MS subjects from low (EDSS = 1.0–3.5), moderate (EDSS = 4.0–5.5) and high (EDSS = 6.0) disability subgroups. Greater estimate error is evident for patients with higher disability with the generic population model. Similar plots of the estimation error in walking speed estimation for patients of different EDSS scores are shown for the generic population **(B)** and the personalized **(D)** models, directly illustrating the increase in estimation error for the former in patients with higher disability.

We found that the generic healthy volunteer model (*R*-value = 0.85, *p*-value = 4.74 × 10^−10^) (Figure 1A) had a poorer accuracy for measure of walking speed in the clinic and showed greater variance than did the personalized model (*R*-value = 0.98, *p*-value = 1.85 × 10^−22^) (Figure 1B). Errors in estimation of walking speed using the generic model were almost 4-fold greater in the group with highest relative to the lowest disability (Table 2). Bland-Altman plots of differences between the measured and estimated walking speeds highlight a bias toward overestimation with those walking more slowly (Figure 2A) and an overestimate of speed for those with higher disability using the generic model (Figure 2B). The magnitude of errors is large in some instances: the mean error is more than 50% of the mean gait speed for those patients with highest disability. By contrast, personalized models estimated walking speeds with high accuracy across the full range of walking speed (Figure 2C) and disability (Figure 2D) in our study population (Table 2).

**Table 2:** Comparison between the walking estimation performance of a model developed using data from healthy volunteers (Healthy Volunteer Model) and of a personalized model based on subject-specific gait calibration (Personalized Model).

**Group**	**Measured (m/s)**	**Healthy volunteer model (m/s)**		**Personalized model (m/s)**	
		**Estimated**	**Error**	**Estimated**	**Error**
Overall (*n* = 32)	1.05 (0.35)	1.09 (0.21)	0.03 (0.20)	1.04 (0.33)	−0.01 (0.07)
Low (*n* = 20)	1.25 (0.19)	1.17 (0.18)	−0.08 (0.12)	1.22 (0.20)	−0.03 (0.08)
Moderate (*n* = 6)	0.91 (0.32)	1.06 (0.19)	0.16 (0.20)	0.90 (0.27)	−0.01 (0.06)
High (*n* = 6)	0.55 (0.15)	0.83 (0.09)	0.28 (0.12)	0.57 (0.12)	0.02 (0.05)

*The values in the table are mean (± SD). Low, Moderate, and High refers to the MS volunteers with EDSS scores of 1.0–3.5, 4.0–5.5, and 6.0, respectively. Measured refers to the walking speed manually measured by the researcher during the data collection; Estimated values are the walking speeds calculated from actigraphy data using the models. Error expresses the differences between the directly measured and estimated speeds*.

**Figure 3:**
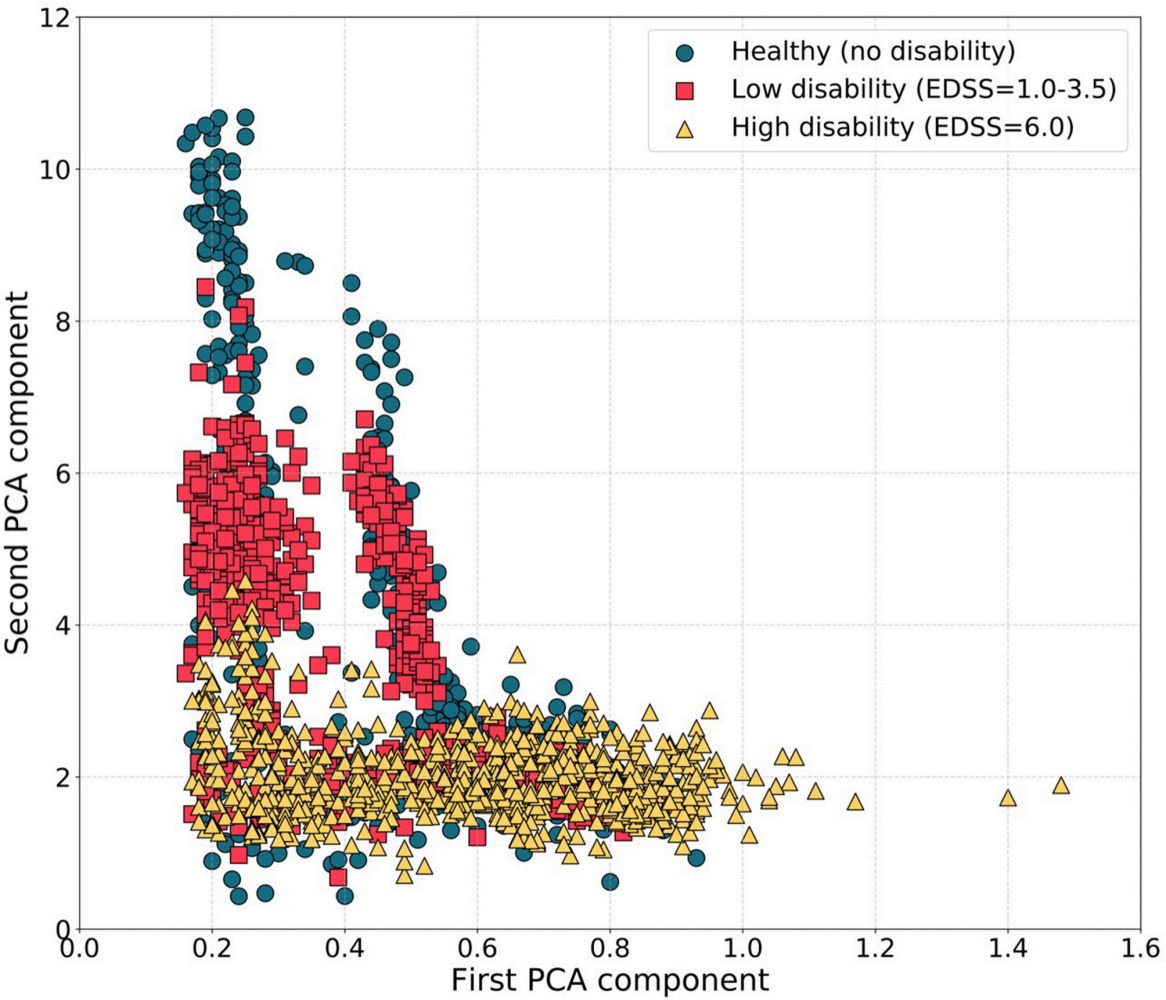
Scatter plots of the first and second PCA components of step features during the T25FW for participants with different disability levels. Principal components for the healthy subjects and MS volunteers with low disability (EDSS 1.0–3.5) were similar and distinct from those for MS volunteers with highest disability.

To explain the relative failure of the generic healthy volunteer model, we tested for differences in step features between the patients and the healthy volunteers using PCA. Principal components for the low disability and healthy volunteer groups overlapped and were distinct from those for the higher disability group, consistent with differences in step patterns between participants in the highest disability group and both healthy volunteers and less disabled patients with multiple sclerosis (Figure 3).

### Home monitoring shows a strong correlation between walking in the home environment and the clinic-based assessment

**Figure 4:**
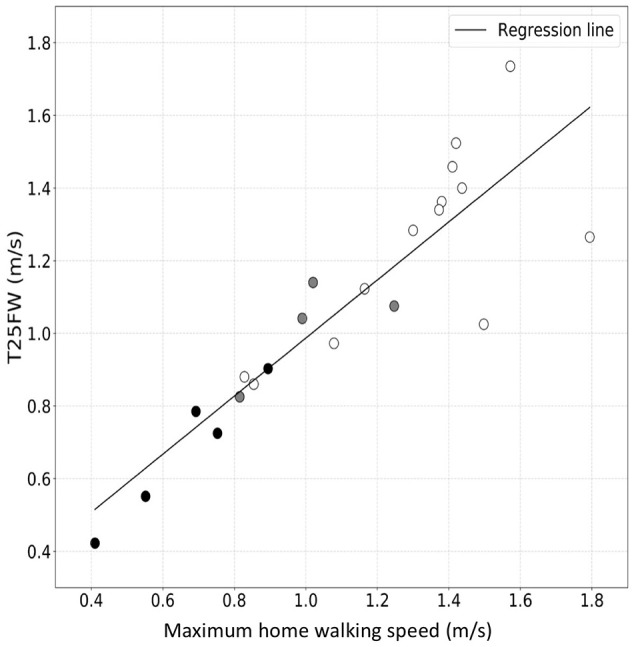
Correlation between maximum sustained walking speed in the home environment and T25FW speed measured in the clinic. The white-filled, gray-filled circles, and black-filled circles denote MS subjects from low (EDSS = 1.0–3.5), moderate (EDSS = 4.0–5.5) and high (EDSS = 6.0) disability subgroups. These results suggest good agreement across the range of disability.

We then used personalized models to estimate the maximum sustained walking speeds in the home environment from the actigraphy measures acquired as the patients went about their usual daily activities in their home environments (Table 3). Maximum sustained walking speeds over a single day are illustrated for Subject 1 in the Supplementary Material (Supplementary Figure 2). We compared maximum sustained gait speeds measured in the home with the T25FW speeds measured in the clinic for each subject to test how well the clinic measure predicts home performance. There was a strong correlation between maximum sustained walking speeds at home and the T25FW walking speed measured in the clinic (*R*-value = 0.89, *p*-value = 4.34 × 10^−8^) (Figure 4). We did not find evidence for a bias in the accuracy of the clinic T25FW prediction of the maximum sustained home walking speed with either gait speed or disability (Supplementary Figure 3).

**Table 3:** Individual Timed 25 Foot Walk speeds directly measured in the clinic (Clinic T25FW Speed, meters/sec) and the maximum (Max home gait speed) and mean of highest (Mean home gait speed) sustained gait speeds estimated remotely in the home environment.

**Patient**	**Clinic T25FW speed (m/s)**	Max home gait speed^*^ (m/s)	Mean home gait speed^*^(m/s)
1	1.5	1.43	1.30
9	1.0	1.11	0.96
10	1.4	1.49	1.32
12	1.0	0.86	0.84
13	1.4	1.38	1.27
14	1.0	0.83	0.81
15	1.1	0.99	0.88
17	1.0	0.99	0.95
18	0.9	0.88	0.81
19	1.3	1.16	1.07
21	1.3	1.24	1.17
22	1.1	1.17	1.07
23	0.5	0.55	0.55
24	0.8	0.66	0.61
25	0.4	0.38	0.38
26	0.7	0.78	0.77
27	1.7	1.57	1.30
28	0.8	0.78	0.71
29	1.1	1.26	1.16
30	1.0	1.42	1.20
31	1.3	1.33	1.26
32	1.4	1.44	1.25

Good agreement was found between the clinic and home remote measures (see section Results).

* *Sustained for ≥7.62 m (25 ft) with the mean values assessed over all such walks identified in the recorded observation periods*.

### The actigraphy device is well tolerated for longer term monitoring in home environments

29/32 participants reported that they felt comfortable while wearing the device for extended periods in both the clinic and home environments. This feedback suggested that device could be worn on the lower back continuously without concern by most subjects for at least a week. Three participants felt uncomfortable wearing the device. One participant reported that the device pressed uncomfortably on the back when sitting on a chair. Another experienced an uncomfortable subjective sensation of “warmth.” The third participant was unable to reposition the device without assistance when at home.

## Discussion

Unlike most prior analyses of actigraphy data, we have focused on deriving absolute measures of gait speed, as opposed to, e.g., measures of numbers of steps taken. We did this because gait speed—and not numbers of steps—is used as a clinical performance measure to assess disability and responses to treatment for people with MS. We have addressed a limitation of previous approaches using population-based models that precludes accurate measures from patients with higher disability. Here we used machine learning to individually calibrate gait models for each patient. We then showed that these personalized models can estimate walking speed accurately from a single tri-axial accelerometer worn in the lower back. Using data from periods of sustained walking while the patients were in their home environment (estimated as 25 feet or greater), we confirmed for the first time that the T25FW measured in the clinic is an accurate index of “real life” maximum sustained walking speed. This provides a first direct validation of the clinical meaningfulness of the T25FW used routinely in the clinic.

There have been several previous studies using mobile actigraphy devices to estimate walking parameters in home environment. These have used commercially available actigraphy systems, such as the Actibelt, Fitbit, and Apple iPhone (4, 21). The devices have been worn in different positions on the body, with agreement that wearing them near the center of gravity near the body mid-line may provide most accurate measures (20, 21). Most of these approaches are distinguished from what we report here by estimating walking speed based on generic population models, which assume that all subjects (i.e., healthy subjects and patients) have similar step and gait characteristics. Here we highlighted that previous approaches cannot provide accurate walking speed estimation for patients with MS over the full range of EDSS because patients with greater disability show differences in step characteristics. Our approach differs from these efforts in that we have used machine learning to generate a *personalized* model for each subject by utilizing individual actigraphic data collected during short calibration walks at the clinic. We also used free walking data for both calibration and validation of the model, instead of treadmill walking, which has been used previously (19).

We placed the Axivity AX-3 device in the lower back, near the body center of mass. This was generally well-tolerated. A recent study using a single, population based model for estimating walking speed on a treadmill for both healthy volunteers and patients with MS directly tested different combinations of the number of actigraphy devices and their arrangements on subjects' body to determine the most informative way of collecting actigraphy data for the estimation of walking speed (19). They confirmed that a single device attached near the midline of body above the pelvis (sacrum or waist) gave the best results.

We acknowledge several limitations of our study. Although there was very good agreement between the clinic and home walking speed measures overall, the four MS volunteers in the lowest disability group (EDSS 1) had higher gait speeds measured in the clinic during T2FW than was estimated as the mean sustained walking speeds at home, although the relative difference is low (6.4% mean standardized difference). We can only speculate why this may have arisen, e.g., Future work could investigate factors influencing the predictive accuracy of clinical walking performance measures. We also did not attempt to assess the sensitivity of the remote monitoring gait speed estimates to changes over time or the relationship to activities of daily living.

We believe that, in addition to regular updates of walking actigraphy data on patients, their personalized gait models likely will need periodic re-calibration, as gait characteristics will change with changes in disability (Supplementary Figure 2). Future work should assess the relative sensitivity of both walking speed and step characteristics to changes over time across a range of disability. Future work also could explore relationships between these walking speed measures and self-report questionnaires on activities of daily living (29) to better understand the clinical meaningfulness of changes in step characteristics and gait speeds. These results could be related directly to effects of treatments. A final limitation of our study lies in the singular focus on the accuracy of walking speed estimates. Additional, potentially clinically meaningful measures also could be extracted from these and similar data. For example, frequencies of periods of activity can be assessed and correlates of night time sleep can be extracted. These topics all could be addressed in future work.

In summary, we have demonstrated that individualized modeling and calibration can accurately estimate walking speed remotely independent of disability level in patients with MS. We showed the feasibility and tolerability of wearing the accelerometer on the lower-back position for acquisition of the most informative data over several days of home gait monitoring. Using these methods, we provided a novel validation of the T25FW test by demonstrating its strong correlation with the maximum sustained free walking speed in the home environment. Future work needs to explore whether continuous home monitoring of gait speed provides a better index of treatment responses than does the sparsely sampled clinic measures or a more sensitive measure of disability progression over time.

## Author contributions

AS: acquisition of data, analysis and interpretation of data. GD and AG: acquisition of data. RN: study concept and design, acquisition of data. YG: study concept and design, analysis and interpretation of data. PM: study concept and design, analysis and interpretation of data, study supervision.

### Conflict of interest statement

The authors declare that the research was conducted in the absence of any commercial or financial relationships that could be construed as a potential conflict of interest.
