# Supplementary material for: Remote Monitoring in the Home Validates Clinical Gait Measures for Multiple Sclerosis
Source: Front Neurol. 2018 Jul 13;9:561. doi: 10.3389/fneur.2018.00561 (PMC6053510; doi:10.3389/fneur.2018.00561)
Supplement: Supplementary file 1 [file Data_Sheet_1.docx]

**Supratak et al. Supplementary Material**

**Supplementary Methods**

# *Calibr**ation of a personalised gait model*

Support Vector Regression (SVR)^25^ was used to build a personalized model that estimates walking speed from the input step features. Formally, suppose there are $m$ step data annotated with their corresponding walking speed $\left\{ \left( \boldsymbol{x}^{\boldsymbol{(1)}},y^{(1)} \right),\ldots,\left( \boldsymbol{x}^{(i)},y^{(i)} \right),\ldots,\left( \boldsymbol{x}^{(m)},y^{(m)} \right) \right\}$ in the training data, where $\boldsymbol{x}^{(i)}$ is the $i$-th step feature, $\boldsymbol{x}^{(i)}\in\mathbb{R}^{n}$, $n$ is the number of step features (which is 29 in this study), and $y^{(i)}$ is a floating-point number of the corresponding walking speed (i.e., ground-truth). The SVR model represents a function that estimates a walking speed for each step data $x^{(i)}$ as follows:

$$\hat{y}^{(i)}=\text{SVR}_{\theta}\left( \boldsymbol{x}^{(i)} \right)$$

where $\hat{y}^{(i)}$ is the estimated walking speed of $\boldsymbol{x}^{(i)}$, and $\theta$ is the $\text{SVR}$ model parameters.

During the model calibration, the parameters $\theta$ are optimized using the training data such that the $\text{SVR}$ model can estimate $\hat{y}^{(i)}$ that deviates from $y^{(i)}$ by a value no greater than an epsilon $\varepsilon$ for each $\boldsymbol{x}^{(i)}$. This calibration process stops when it has reached convergence. The hyperparameters of the $\text{SVR}$ model such as kernel function, $C$ and $\varepsilon$ are chosen via a grid search with 10-fold cross-validation on the training data. The best hyperparameters are the ones that give the lowest mean-squared-error (MSE), and they vary across subjects. Supplementary Table 3 shows a range of the hyperparameters supplied during the model calibration.

After the model calibration, the model was applied to the upcoming acceleration data from the same subject. Our model estimated the walking speed at each step; the speed can be calculated as the average of the speed estimated from all steps during such walking period.

Our rationale for using a model that estimates walking speed for each step is that people can walk at different speeds during a period of sustained walking. Also, splitting a sustained walk into steps simplified the feature extraction process allowing us to derive interpretable features from the step data.

# *Development of a healthy volunteer gait speed model*

To highlight potential limitations of use of a generic (rather than a personalized) model for estimation of gait speed from actigraphy data, the same SVR method was used to calibrate a model from grouped data collected previously from healthy volunteers. Data from 7 healthy subjects (6 men, 1 woman, mean age, 29.7 ± 3.9 years) asked to walk 25 feet at three different speeds (slow, normal and fast) at least 12 times was used. Times for each walk were recorded for calculation of walking speeds. The mean ± standard-deviation (SD) of the slow, normal and fast speeds were 0.63 ± 0.10, 1.10 ± 0.15, and 1.62 ± 0.30 m/s, respectively. These walking data and their corresponding walking speeds were used together to construct a healthy volunteer population model based on the method above.

**Supplementary Tables**

**Supplementary Table 1. 2 min walk times and calculated gait speeds for patients in the study.**

| **Patient** | **EDSS** | **Age** | **Gender** | **Distance (m)** | **Gait Speed 2-min (m/s)** |
| --- | --- | --- | --- | --- | --- |
| 1 | 2 | 45 | M | 163.5 | 1.4 |
| 2 | 2 | 30 | F | 140.2 | 1.2 |
| 3 | 5.5 | 43 | M | 91.3 | 0.8 |
| 4 | 1 | 30 | F | 158.4 | 1.3 |
| 5 | 5 | 47 | M | 70.7 | 0.6 |
| 6 | 2.5 | 33 | M | 151.0 | 1.3 |
| 7 | 3 | 37 | F | 134.3 | 1.1 |
| 8 | 2.5 | 36 | F | 138.3 | 1.2 |
| 9 | 3 | 53 | F | 142.6 | 1.2 |
| 10 | 2 | 42 | M | 187.3 | 1.6 |
| 11** | 6 | 48 | F | 53.6 | 0.5 |
| 12 | 1 | 41 | F | 114.1 | 1.0 |
| 13 | 2 | 29 | M | 167.5 | 1.4 |
| 14 | 1.5 | 34 | M | 110.0 | 0.9 |
| 15 | 4 | 46 | F | 152.0 | 1.3 |
| 16 | 3.5 | 52 | M | 140.0 | 1.2 |
| 17 | 4 | 35 | F | 135.6 | 1.1 |
| 18 | 6 | 54 | F | 125.4 | 1.1 |
| 19 | 2 | 33 | F | 110.1 | 0.9 |
| 20 | 1 | 46 | F | 150.5 | 1.3 |
| 21 | 2.5 | 39 | F | 173.8 | 1.5 |
| 22 | 1.5 | 32 | F | 155.1 | 1.3 |
| 23 | 6 | 47 | F | 153.5 | 1.3 |
| 24 | 6 | 41 | M | 72.2 | 0.6 |
| 25 | 6 | 49 | M | 122.1 | 1.0 |
| 26 | 6 | 59 | M | 84.6 | 0.7 |
| 27** | 1 | 28 | F | 55.8 | 0.4 |
| 28 | 5.5 | 32 | M | 92.6 | 0.8 |
| 29 | 5.5 | 35 | F | 185.1 | 1.5 |
| 30 | 1.5 | 28 | M | 110.2 | 0.9 |
| 31 | 1 | 29 | M | 160.6 | 1.3 |
| 32 | 1 | 45 | M | 184 | 1.5 |

* Patient 11 stopped after 113.6 seconds due to fatigue.

** Patient 27 limited the walk to one trial due to fatigue. She completed this trial in 128.0 seconds.

**Supplementary Table 2. The 29 features derived by our personalised model from data for each actigraphy data associated with each step.**

| **Feature** | **Equation** | **Dimension of acceleration to compute features** |
| --- | --- | --- |
| Mean | $\frac{\sum_{i=1}^{n} s_{i}}{n}$ | $energy$ acceleration |
| Variance | $\frac{{\sum_{i=1}^{n} \left( s_{i}-mean \right)}^{2}}{n}$ | $ver$, $hor$, $fwd$, and $energy$ acceleration |
| Maximum | $\max(s_{1},\ldots,s_{n})$ | $ver$, $hor$, $fwd$, and $energy$ acceleration |
| Minimum | $\min(s_{1},\ldots,s_{n})$ | $ver$, $hor$, $fwd$, and $energy$ acceleration |
| Range | $\mathrm{Maximum}-\mathrm{Minimum}$ | $ver$, $fwd$, and $energy$ acceleration |
| Maximum-mean | $Maximum-Mean$ | $ver$, $hor$, $fwd$, and $energy$ acceleration |
| Step amplitude | $\frac{Maximum-Mean}{n}$ | $ver$, $hor$, $fwd$, and $energy$ acceleration |
| Root mean square (RMS) | $\sqrt{\frac{\sum_{i=1}^{n} s_{i}^{2}}{n}}$ | $ver$, $hor$, $fwd$, and $energy$ acceleration |
| Step duration | $n/F_{s}$ | Number of sample from any dimension |
| **Note**: Each step data consists of $n$ values of $s_{1},\ldots,s_{n}$ from three dimensions: vertical ($ver$), horizontal ($hor$) and forward ($fwd$). $energy$ acceleration can be computed from $e_{i}=\sqrt{ver_{i}^{2}+{hor}_{i}^{2}+{fwd}_{i}^{2}}$. $F_{s}$ is the sampling rate of recording device. | | |

**Supplementary Table 3. Ranges of hyperparameters of the Support Vector Regression model used for personalized model calibrations.**

| **Hyperparameter** | **Range** |
| --- | --- |
| Kernel | $\left\{ \text{Linear}, \text{Gaussian} \right\}$ |
| C | $\left\{ 2^{-8},2^{-6},\ldots,2^{8} \right\}$ |
| epsilon ($\varepsilon$) | 0.01 |
| gamma ($\gamma$) (for Gaussian kernel) | $\left\{ 2^{-8},2^{-6},\ldots,2^{8} \right\}$ |

**Supplementary Figure Captions**

**Supplementary Figure 1. An example of raw 3D acceleration data collected from the accelerometer worn by a patient with MS (subject 1), illustrating alternating vertical (red), horizontal (green) and forward (blue) acceleration through serial steps.** Each set of 3 points along the abscissa describes the data from the 3 accelerometers in the device that is acquired at a single time. A single step cycle is segmented by the rectangle in the graph to the left. During data pre-processing, each step cycle is segmented and serially aligned, as illustrated to the right. Features described in Supplementary Table 2 are extracted from these automatically by the analysis algorithm.

**Supplementary Figure 2. Examples of estimated walking shows the walking speeds estimated for sustained (> 25 foot) walks over two consecutive days (A,B) for subject 1.**

**Supplementary Figure 3. Testing for systemic bias in agreement between clinic T25FW and home walking speeds.** First, we assessed the difference between the mean maximum sustained walking speeds **(MAX-SUS-HOME)**  estimated remotely in the home environment and the mean clinic T25FW speed using a with Bland-Altman plot (A). The differences did not appear to change with walking speed. Second, we tested variation of differences between home walking speed and the T25FW with EDSS in our population (B). No significant change was seen, consistent with clinical meaningfulness of the EDSS measure across the disability range
