# Supplementary figures and images for: Remote Monitoring in the Home Validates Clinical Gait Measures for Multiple Sclerosis
Source: Front Neurol. 2018 Jul 13;9:561. doi: 10.3389/fneur.2018.00561 (PMC6053510; doi:10.3389/fneur.2018.00561)

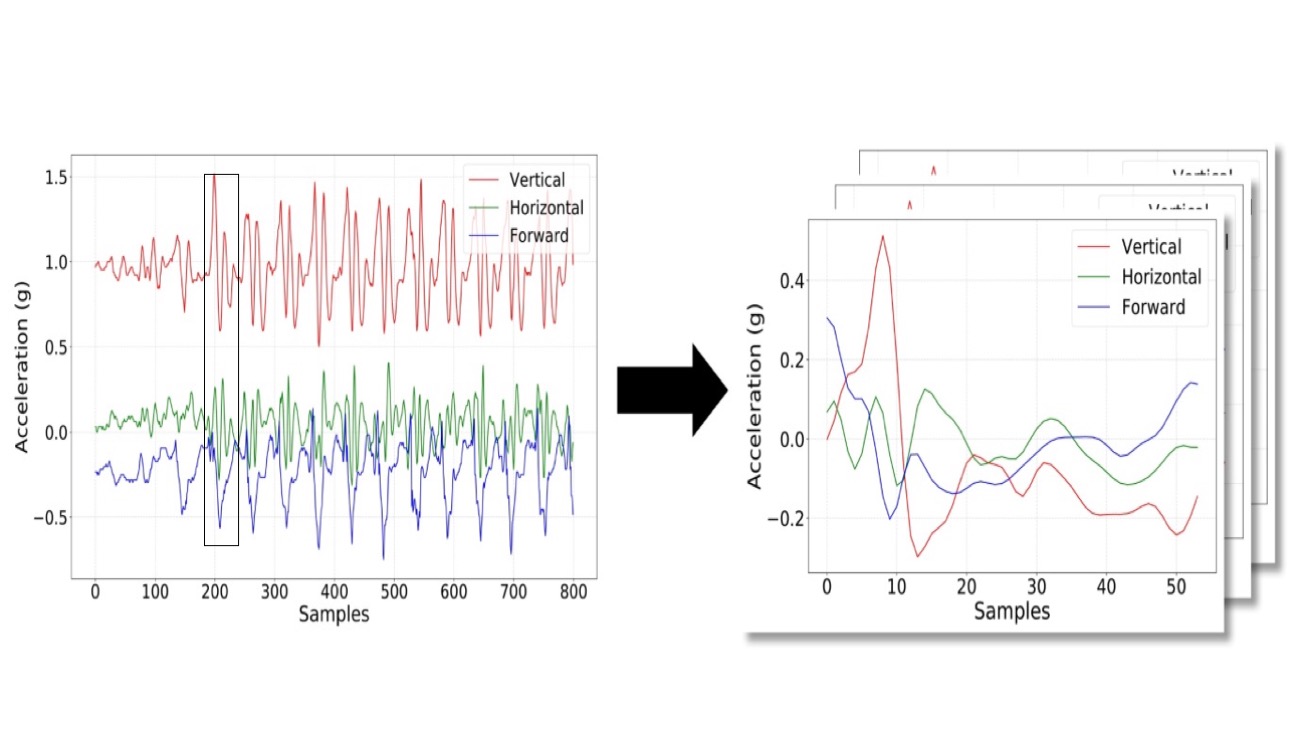

Supplement: Supplementary file 2 [file Image_1.JPEG]

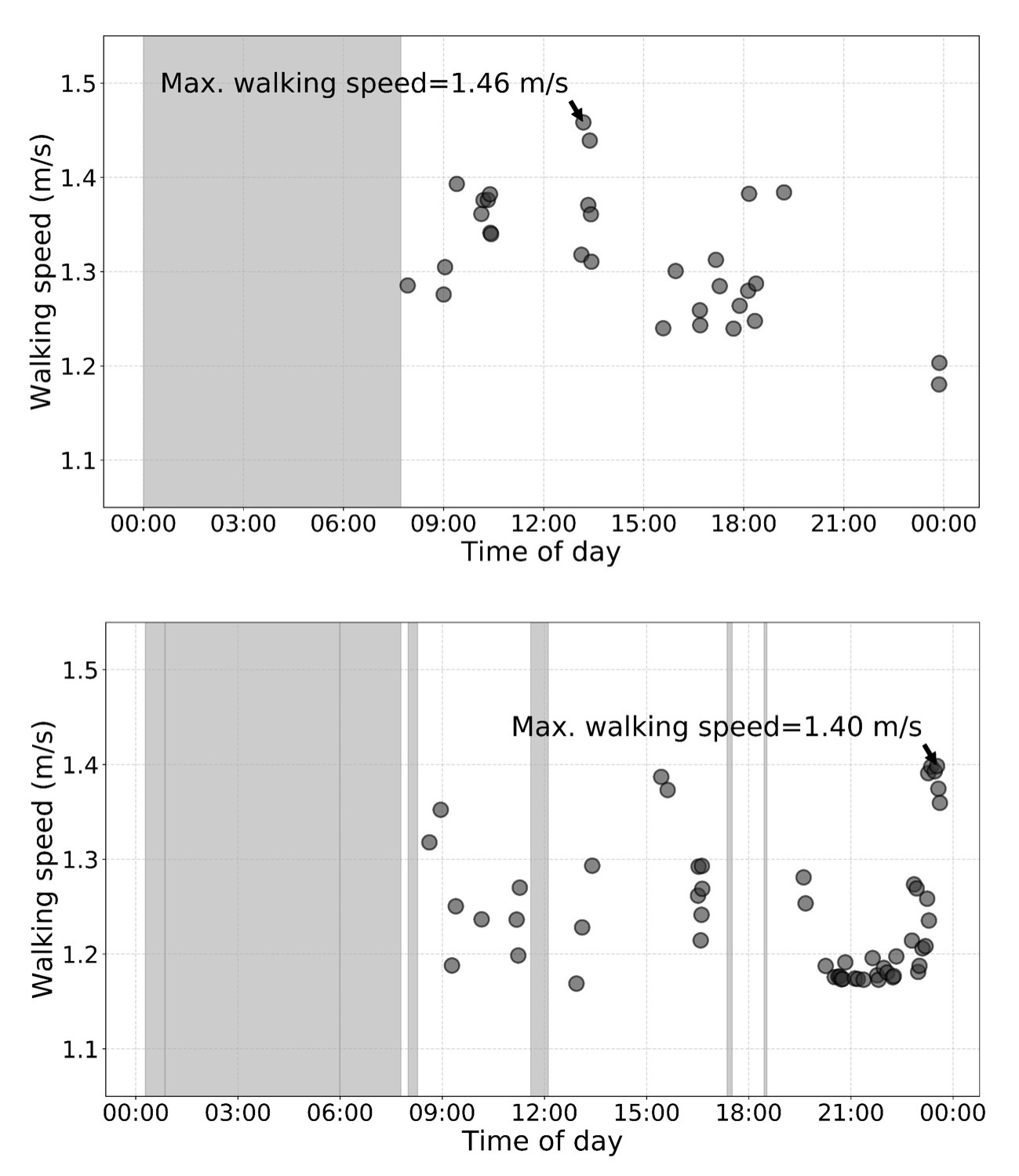

Supplement: Supplementary file 3 [file Image_2.JPEG]

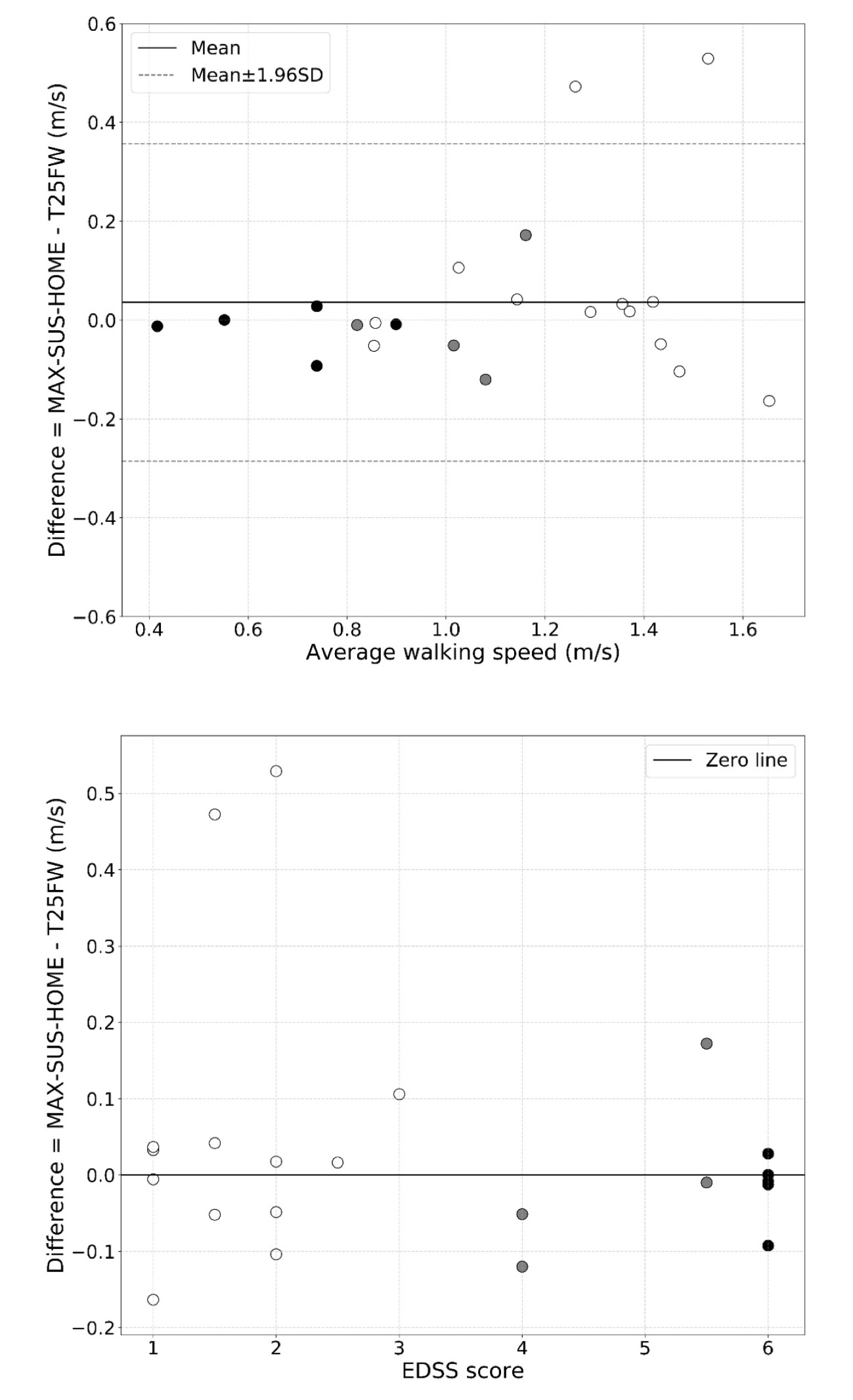

Supplement: Supplementary file 4 [file Image_3.JPEG]
